# Supplementary material for: Neural mechanisms for the localization of unexpected external motion
Source: Nat Commun. 2023 Sep 30;14:6112. doi: 10.1038/s41467-023-41755-z (PMC10542789; doi:10.1038/s41467-023-41755-z)
Supplement: Supplementary file 1 — Supplementary Information [file 41467_2023_41755_MOESM1_ESM.pdf]

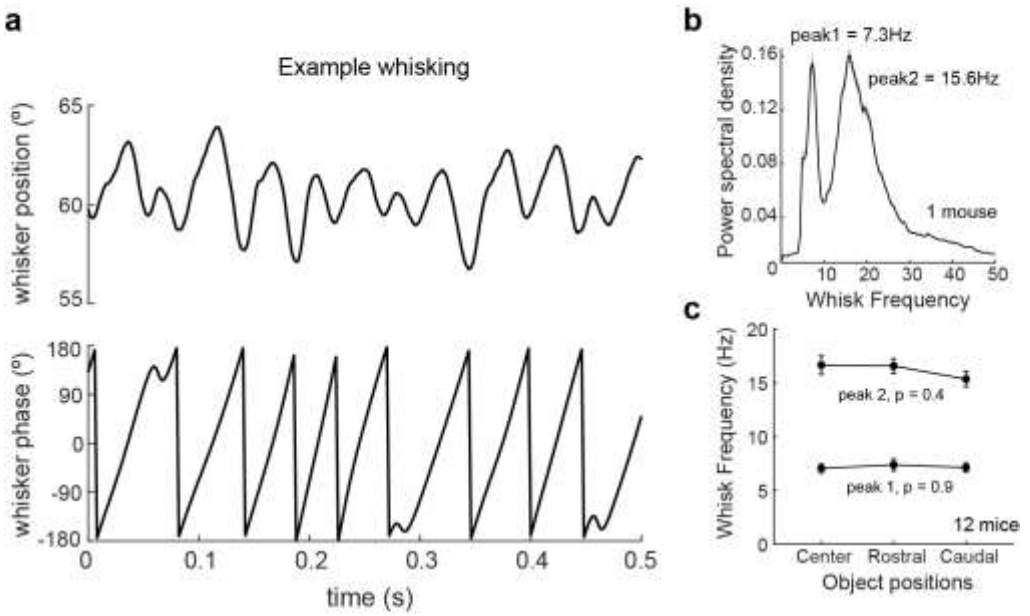

Supplementary Figure 1. Whisk frequency was stable across stimulus conditions

a) Whisker position and phase during a sample period. **b**) Power spectral density of whisk frequency calculated using whisker phase (1 mouse). Notice how the distribution has two prominent peaks **c**) Mean whisk frequency as a function of object position, calculated for each peak in the power spectrum (1-way anova,12 mice). Error bars represent s.e.m. All values are mean ± s.e.m. Source data are provided as a Source Data file.

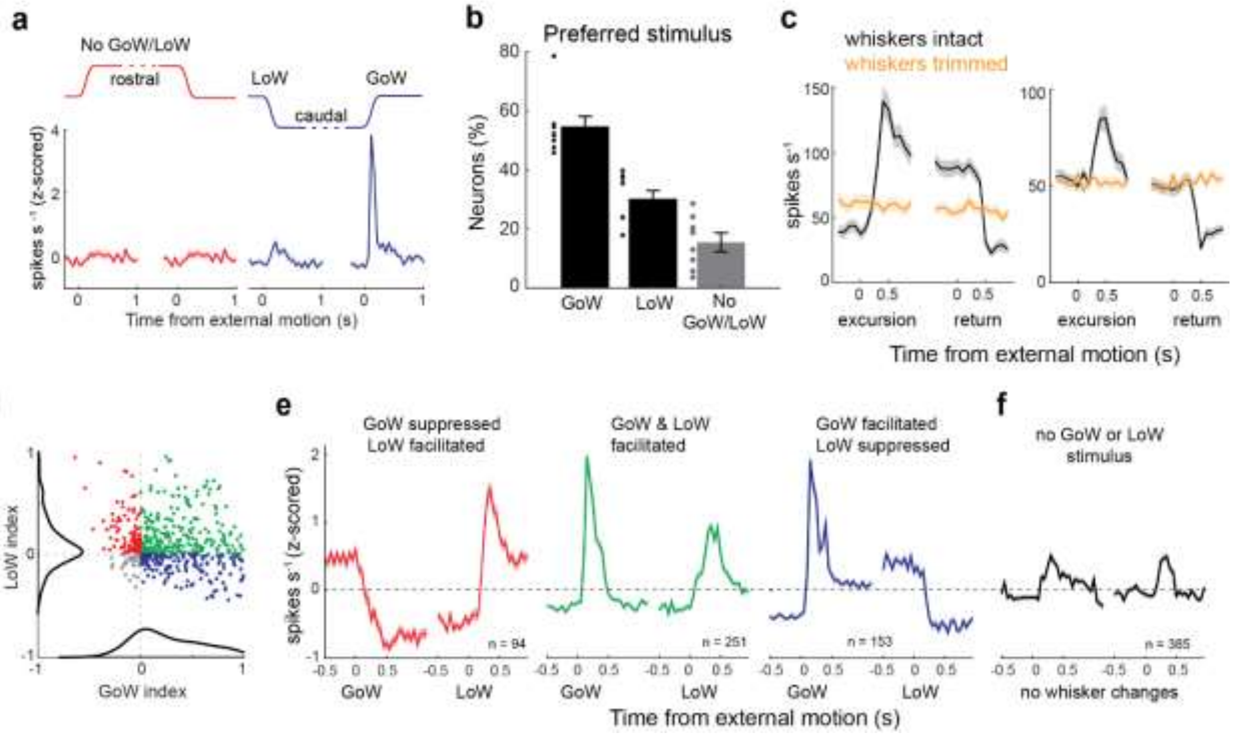

Supplementary Figure 2. Gains and losses in whisker contact during external motion elicit the transient response

a) Population firing rates from an example mouse in Fig. 2A during surface movements. A gain/loss in whisker contact only occurred during external movement in the caudal space. (46 neurons) **b)** Stimulus preference of neurons to gain of whisker, loss of whisker or no change in whisker contact (385 neurons from 8 mice). Error bars represent s.e.m. **c)** The trial-averaged firing rate of two example neurons with intact whiskers and after all the whiskers were trimmed off. **d)** Scatter plot comparing the GoW and LoW modulation of each neuron (10 mice, 529 neurons). **e)** Population averaged firing rates of neurons grouped by the sign (+/-) of their responses to GoW and LoW (8 mice, neurons). **f)** Population averaged firing rates in response to surface movement without GoW or LoW stimulation (8 mice, 385 neurons). All population average firing rates are binned at 50ms. All values are mean ± s.e.m. Source data are provided as a Source Data file.

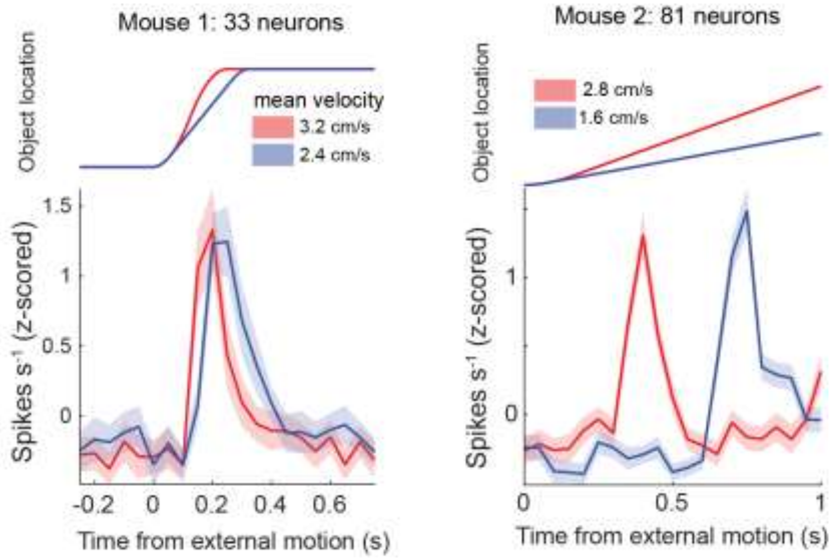

Supplementary Figure 3. Moderate differences in external motion velocity did not alter transient response magnitude

Population averaged firing rates in two mice during GoW stimulation at two different external motion velocities. Notice how response magnitude is the same, but response onset is sooner for faster velocities, due to GoW stimulation occurring earlier in time. All firing rates are binned at 50ms. All values are mean ± s.e.m. Source data are provided as a Source Data file.

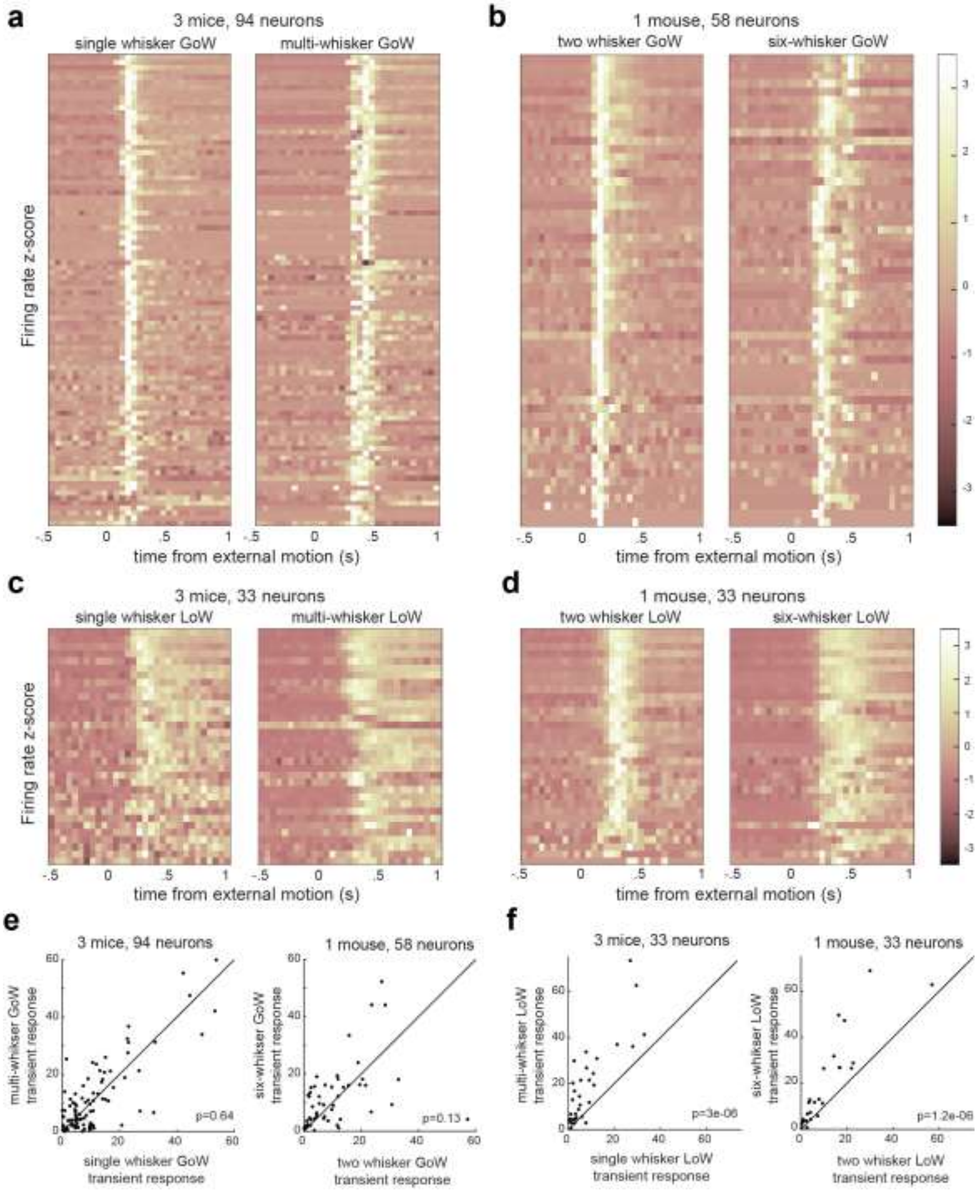

Supplementary Figure 4. Single and multi-whisker gains in whisker contact generate similar transient responses overall

a, b) Firing rates of SC neurons during single and multi-whisker GoW stimulation. **c,d**) Firing rates of SC neurons during single and multi-whisker LoW stimulation. **e, f**) Scatter plots comparing neuronal responses to single and multi-whisker stimulation (**e** – GoW stimulus; **f** – LoW stimulus). A paired, two-sided Wilcoxon signed rank test was used as a statistical test in e and f. Source data are provided as a Source Data file.

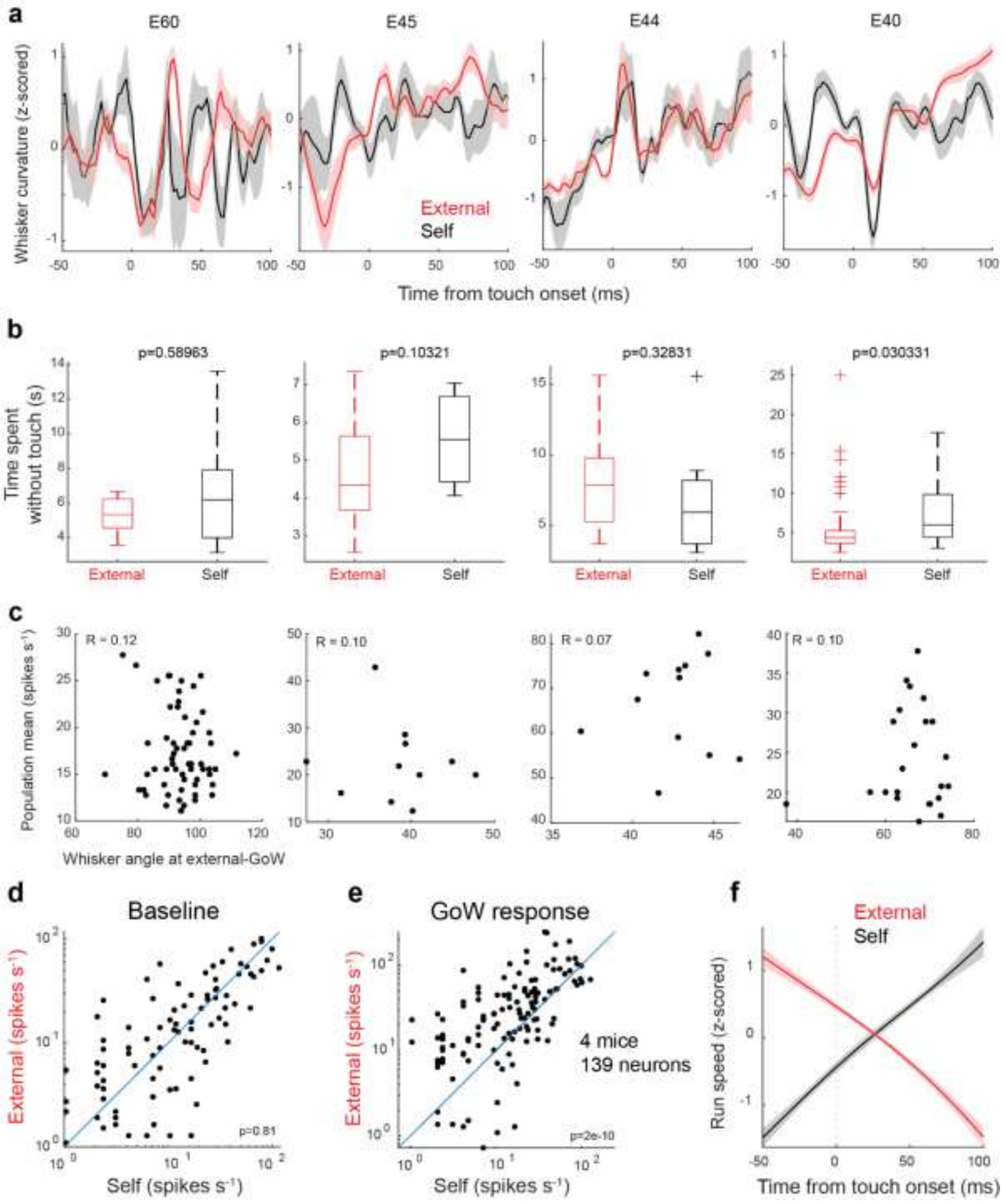

Supplementary Figure 5. Whisker curvature, repetition rate, angle of touch and baseline activity do not explain the larger external-GoW response

a) Trial-averaged whisker curvature aligned to the onset of touch during external and self-generated touch events. Each plot is from a separate animal (four total) **b)** The amount of time each animal spent without touch before experiencing an external or self-generated touch event. Each plot is from a separate animal (n=23,13,11,61 external touches; n=7,8,7,16 self-touches, p

values are from two-sided Mann Whitney U-test). The central mark indicates the median. The bottom and top edges of the box indicate the 25th and 75th percentiles, respectively. The bottom and the top edges of the whiskers are the minima and maxima excluding outliers. '+' indicates outliers. **c**) No correlation between whisker angle at the moment of extenal-GoW contact and the mean firing rate of SC neurons, as demonstrated in four mice. Pearson correlation coefficient values for each mouse are displayed on the top of the scatter plot. **d, e**) Scatter plots of baseline and GoW firing rates during self- and external-GoW stimulation (two-sided Wilcoxon signed rank test, $p = 0.81$ for pre-touch, $p=2e^{-10}$ for touch response, 4 mice, 139 neurons). **f**) Mouse run speed during external and self-generated stimulation. All values are mean ± s.e.m. Source data are provided as a Source Data file.

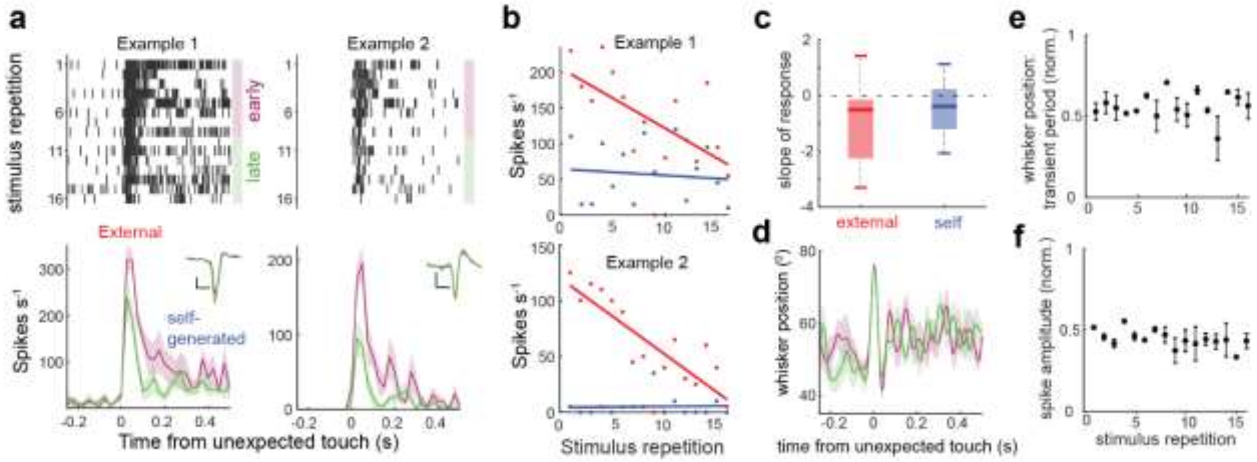

Supplementary figure 6. **Habituation of the transient response to externally generated touch**

a) Rasters and histograms of spiking in two example SC neurons around the onset of external-GoW stimulation. The histograms are divided by early (1st half) and late (2nd half) trials. **b)** Magnitude of the external-GoW and the persistent self-generated responses in these neurons as a function of stimulus repetition. Firing rate as a function of stimulus repetition was fit with a linear regression. **c)** Slope of the linear regression for each response type (40 external responses, $p = 5e^{-4}$;14 self-responses, $p = 0.08$; 2 mice, one-sided t-test). The central mark indicates the median. The bottom and top edges of the box indicate the 25th and 75th percentiles, respectively. The bottom and the top edges of the whiskers are the minima and maxima excluding outliers. **d)** Comparison of whisker position for early (purple) and late (green) trials around the onset of touch. **e)** Normalized whisker position as a function of stimulus repetition (n=2 mice). **f)** Normalized spike waveform amplitude as a function of stimulus repetition (n=2 mice). All values are mean ± s.e.m. Source data are provided as a Source Data file.

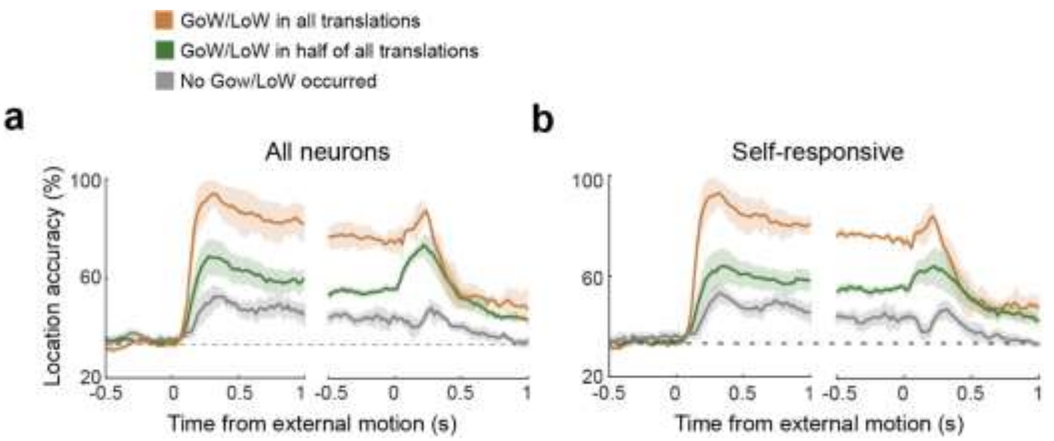

Supplementary figure 7. Persistent self-generated neural code for surface location

a) Decoding accuracy using all recorded neurons averaged across animals based on changes in whisker contact (GoW/LoW in all movements: 2 mice, 81 neurons; GoW/LoW in half of all movements: 6 mice, 262 neurons; zero GoW/LoW movements: 4 mice, 149 neurons). **b)** Decoding accuracy for self-responsive neurons only (GoW/LoW in all movements: 2 mice, 22 neurons; GoW/LoW in half of all movements: 6 mice, 166 neurons; Zero GoW/LoW movements: 4 mice,116 neurons). All values are mean ± s.e.m. Source data are provided as a Source Data file.

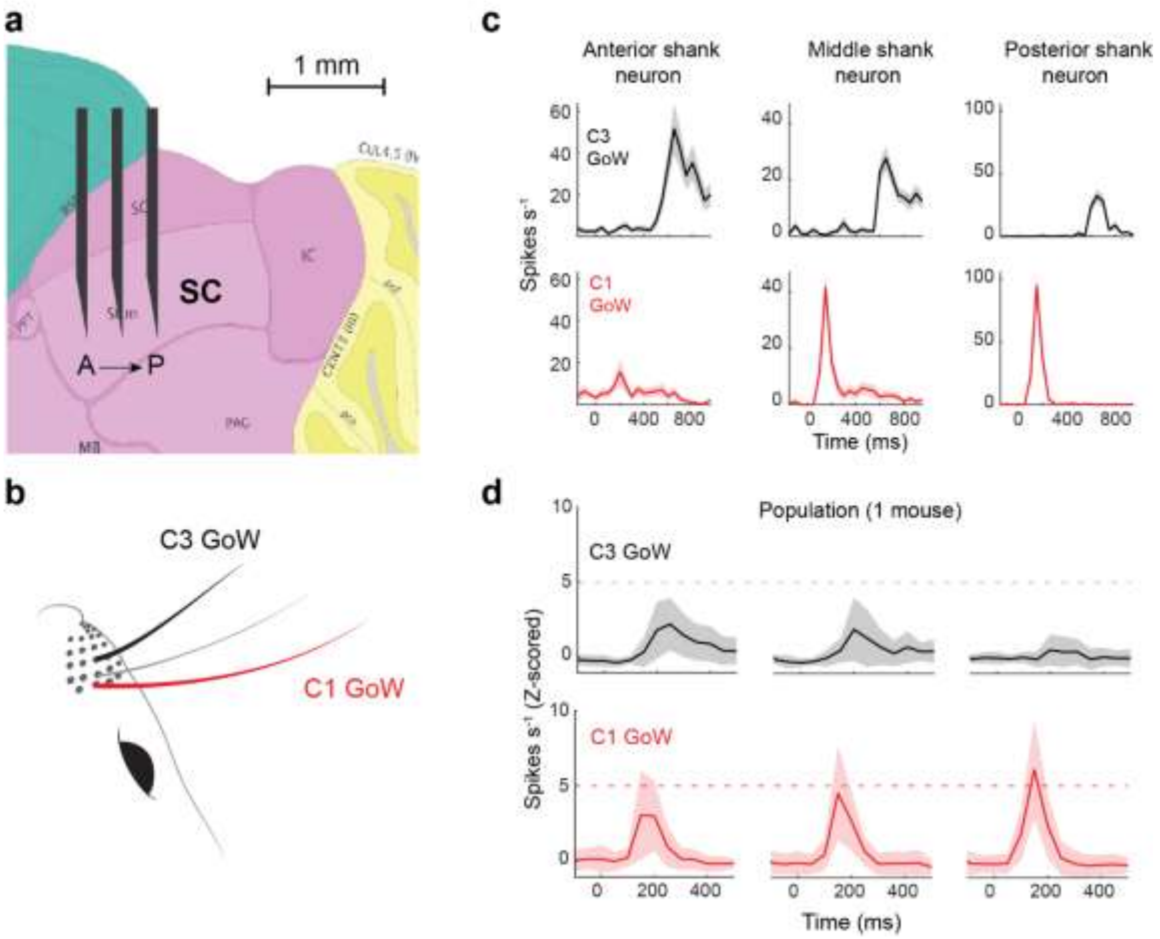

Supplementary figure 8. The somatotopic organization of whisker space

a) Orientation of electrode shanks in the SC. **b)** Illustration of whisker stimulation that generates neural responses in panel C and D. C1 GoW stimulation is represented in red, C3 GoW stimulation is in black. Anatomical annotations from the Allen Mouse Brain Atlas[1] and Allen Reference Atlas – Mouse Brain[2]. Allen Mouse Brain Atlas, mouse.brain-map.org and atlas.brain-map.org. **c)** One example neuron from each shank arranged from anterior to posterior in the SC. **d)** Population averaged firing rates of neurons from 3 shanks (30, 23, 43 neurons per shank, from anterior to posterior, 1 mouse). All values are mean ± s.e.m. Source data are provided as a Source Data file.

Supplementary References

1. Allen Institute for Brain Science (2004). Allen Mouse Brain Atlas [dataset]. Available from mouse.brain-map.org. Allen Institute for Brain Science (2011).

2. Allen Reference Atlas – Mouse Brain [brain atlas]. Available from atlas.brain-map.org.
