# Supplementary material for: Neural mechanisms for the localization of unexpected external motion
Source: Nat Commun. 2023 Sep 30;14:6112. doi: 10.1038/s41467-023-41755-z (PMC10542789; doi:10.1038/s41467-023-41755-z)
Supplement: Supplementary file 3 — Description of Additional Supplementary Files [file 41467_2023_41755_MOESM3_ESM.pdf]

File name: Supplementary Movie 1

Description: Rostral Excursion of surface. Example high-speed video clip showing a locomoting mouse whisking against the surface during a translation from center space into rostral space. The mouse is facing the bottom of the frame. The colored dots represent the tracked points of the whiskers that were used to extract kinematic variables.

File name: Supplementary Movie 2

Description: Rostral Return of surface. Example high-speed video clip showing a locomoting mouse whisking against the surface during a translation from rostral space back into center space. The mouse is facing the bottom of the frame. The colored dots represent the tracked points of the whiskers that were used to extract kinematic variables.

File name: Supplementary Movie 3

Description: Caudal Excursion of surface. Example high-speed video clip showing a locomoting mouse whisking against the surface during a translation from center space into caudal space. The mouse is facing the bottom of the frame. The colored dots represent the tracked points of the whiskers that were used to extract kinematic variables.

File name: Supplementary Movie 4

Description: Caudal Return of surface. Example high-speed video clip showing a locomoting mouse whisking against the surface during a translation from caudal space back into center space. The mouse is facing the bottom of the frame. The colored dots represent the tracked points of the whiskers that were used to extract whisker kinematic variables.
