# Supplementary material for: Neural mechanisms for the localization of unexpected external motion
Source: Nat Commun. 2023 Sep 30;14:6112. doi: 10.1038/s41467-023-41755-z (PMC10542789; doi:10.1038/s41467-023-41755-z)
Supplement: Supplementary file 8 — Reporting Summary [file 41467_2023_41755_MOESM8_ESM.pdf]

nature portfolio

Corresponding author(s): Scott R. Pluta

Last updated by author(s): Aug 5, 2023

Reporting Summary

Nature Portfolio wishes to improve the reproducibility of the work that we publish. This form provides structure for consistency and transparency in reporting. For further information on Nature Portfolio policies, see our Editorial Policies and the Editorial Policy Checklist.

Statistics

For all statistical analyses, confirm that the following items are present in the figure legend, table legend, main text, or Methods section.

n/a	Confirmed	
☐	☒	The exact sample size (*n*) for each experimental group/condition, given as a discrete number and unit of measurement
☐	☒	A statement on whether measurements were taken from distinct samples or whether the same sample was measured repeatedly
☐	☒	The statistical test(s) used AND whether they are one- or two-sided<br>*Only common tests should be described solely by name; describe more complex techniques in the Methods section.*
☐	☒	A description of all covariates tested
☐	☒	A description of any assumptions or corrections, such as tests of normality and adjustment for multiple comparisons
☐	☒	A full description of the statistical parameters including central tendency (e.g. means) or other basic estimates (e.g. regression coefficient) AND variation (e.g. standard deviation) or associated estimates of uncertainty (e.g. confidence intervals)
☐	☒	For null hypothesis testing, the test statistic (e.g. *F*, *t*, *r*) with confidence intervals, effect sizes, degrees of freedom and *P* value noted<br>*Give P values as exact values whenever suitable.*
☒	☐	For Bayesian analysis, information on the choice of priors and Markov chain Monte Carlo settings
☒	☐	For hierarchical and complex designs, identification of the appropriate level for tests and full reporting of outcomes
☐	☒	Estimates of effect sizes (e.g. Cohen's *d*, Pearson's *r*), indicating how they were calculated

Our web collection on statistics for biologists contains articles on many of the points above.

Software and code

Policy information about availability of computer code

Data collection	Custom code written in MATLAB 2018a was used to generate trial structure and collect data. Code from Zaber Motion Library (ASCII) was used to control the tactile surface translations .
Data analysis	MATLAB 2019a was used for all data analysis, The Neural Decoding Toolbox (http://www.readout.info/) for classification analysis, Kilosort 2.0 (https://github.com/MouseLand/Kilosort) and phy 2.0 (https://github.com/cortex-lab/phy) for spike sorting and DeepLabCut 2.1 (https://github.com/DeepLabCut) for whisker tracking

For manuscripts utilizing custom algorithms or software that are central to the research but not yet described in published literature, software must be made available to editors and reviewers. We strongly encourage code deposition in a community repository (e.g. GitHub). See the Nature Portfolio guidelines for submitting code & software for further information.

Data

Policy information about availability of data

All manuscripts must include a data availability statement. This statement should provide the following information, where applicable:

- Accession codes, unique identifiers, or web links for publicly available datasets
- A description of any restrictions on data availability
- For clinical datasets or third party data, please ensure that the statement adheres to our policy

The data that supports the findings of this study have been deposited in the DANDI database under accession code 0.230806.003490. Source data are provided with this paper.

Research involving human participants, their data, or biological material

Policy information about studies with human participants or human data. See also policy information about sex, gender (identity/presentation), and sexual orientation and race, ethnicity and racism.

Reporting on sex and gender	Human research participants were not involved in this study.
Reporting on race, ethnicity, or other socially relevant groupings	Human research participants were not involved in this study.
Population characteristics	Human research participants were not involved in this study.
Recruitment	Human research participants were not involved in this study.
Ethics oversight	Human research participants were not involved in this study.

Note that full information on the approval of the study protocol must also be provided in the manuscript.

Field-specific reporting

Please select the one below that is the best fit for your research. If you are not sure, read the appropriate sections before making your selection.

☒ Life sciences ☐ Behavioural & social sciences ☐ Ecological, evolutionary & environmental sciences

For a reference copy of the document with all sections, see nature.com/documents/nr-reporting-summary-flat.pdf

Life sciences study design

All studies must disclose on these points even when the disclosure is negative.

Sample size	No statistical methods were used to predetermine sample sizes. The sample sizes used in our study are considered adequate and consistent with the literature in the field. (E.g. 10.1038/ncomms9114; 10.1038/ncomms14263; 10.7554/eLife.50678) Sample sizes were chosen to measure experimental parameters while remaining in compliance with ethical guidelines for minimizing animal use.<br>For majority of finding, we used data from at least eight animals. For experiments in Figure 3 & Figure 4 where it is impossible to control the behavior of mice in generating self motion, a total of 6 mice were used.
Data exclusions	Only neurons with a significant tactile responses were used in the analyses.
Replication	Replication was done across trials and animals. The exact number of neurons and animal replicates are stated in the figure legends. All results were consistent across animals.
Randomization	In all experiments the tactile stimuli were presented in a randomized order.
Blinding	Investigators were not blinded. Blinding was not necessary for the study, since experimental progress was regulated by the animal's own volition and unbiased by the observer.

Reporting for specific materials, systems and methods

We require information from authors about some types of materials, experimental systems and methods used in many studies. Here, indicate whether each material, system or method listed is relevant to your study. If you are not sure if a list item applies to your research, read the appropriate section before selecting a response.

Materials & experimental systems

n/a	Involved in the study
☒	☐ Antibodies
☒	☐ Eukaryotic cell lines
☒	☐ Palaeontology and archaeology
☐	☒ Animals and other organisms
☒	☐ Clinical data
☒	☐ Dual use research of concern
☒	☐ Plants

Methods

n/a	Involved in the study
☒	☐ ChIP-seq
☒	☐ Flow cytometry
☒	☐ MRI-based neuroimaging

Animals and other research organisms

Policy information about studies involving animals; ARRIVE guidelines recommended for reporting animal research, and Sex and Gender in Research

Laboratory animals	Mice of a CD-1 background of both sexes, between the ages of 9-15 weeks were used for all experiments. Mice were housed at temperatures between 70 – 75 degrees Fahrenheit with a humidity between 40-60%.
Wild animals	No wild animals were used in this study
Reporting on sex	Both male and female mice were used in this study.
Field-collected samples	This study did not involve samples collected from the field.
Ethics oversight	All procedures performed in the study were approved by Purdue Institutional Animal Care and Use Committee (IACUC,1801001676A004) and the Laboratory Animal Program (LAP)

Note that full information on the approval of the study protocol must also be provided in the manuscript.
